# Laboratory contamination in airway microbiome studies

**DOI:** 10.1186/s12866-019-1560-1

**Published:** 2019-08-14

**Authors:** Christine Drengenes, Harald G. Wiker, Tharmini Kalananthan, Eli Nordeide, Tomas M. L. Eagan, Rune Nielsen

**Affiliations:** 1 Department of Thoracic Medicine, Haukeland University Hospital, Bergen, Norway; 2 Department of Clinical Science, Faculty of Medicine, University of Bergen, Bergen, Norway; 3 Department of Microbiology, Haukeland University Hospital, Bergen, Norway

**Keywords:** Microbiome, Contamination, Low biomass, Respiratory, 16S rRNA gene

## Abstract

**Background:**

The low bacterial load in samples acquired from the lungs, have made studies on the airway microbiome vulnerable to contamination from bacterial DNA introduced during sampling and laboratory processing. We have examined the impact of laboratory contamination on samples collected from the lower airways by protected (through a sterile catheter) bronchoscopy and explored various in silico approaches to dealing with the contamination post-sequencing. Our analyses included quantitative PCR and targeted amplicon sequencing of the bacterial 16S rRNA gene.

**Results:**

The mean bacterial load varied by sample type for the 23 study subjects (oral wash>1st fraction of protected bronchoalveolar lavage>protected specimen brush>2nd fraction of protected bronchoalveolar lavage; *p* < 0.001). By comparison to a dilution series of know bacterial composition and load, an estimated 10–50% of the bacterial community profiles for lower airway samples could be traced back to contaminating bacterial DNA introduced from the laboratory. We determined the main source of laboratory contaminants to be the DNA extraction kit (FastDNA Spin Kit). The removal of contaminants identified using tools within the Decontam R package appeared to provide a balance between keeping and removing taxa found in both negative controls and study samples.

**Conclusions:**

The influence of laboratory contamination will vary across airway microbiome studies. By reporting estimates of contaminant levels and taking use of contaminant identification tools (e.g. the Decontam R package) based on statistical models that limit the subjectivity of the researcher, the accuracy of inter-study comparisons can be improved.

**Electronic supplementary material:**

The online version of this article (10.1186/s12866-019-1560-1) contains supplementary material, which is available to authorized users.

## Background

The most common method used for studying the bacterial communities of the lower respiratory tract is high throughput amplicon sequencing of the bacterial 16S ribosomal RNA (16S rRNA) marker gene [1]. Some studies use sputum samples [2, 3], with inevitable questions regarding the degree to which the samples are representative of the lower respiratory tract as opposed to contamination from the upper respiratory tract. The emerging gold standard for lower respiratory tract samples is protected bronchoscopy (sampling via a sterile catheter) [4]. However, even with protected bronchoscopy the samples are processed through extensive laboratory workflows that include at minimum steps of bacterial DNA extraction, PCR amplification of the marker gene, and preparation for sequencing. Each step opens up the possibility for the introduction of contaminating bacterial DNA from the laboratory environment, with greatest impact on samples with the lowest bacterial load [5].

Accurate analysis of the lower respiratory tract microbiome will require separate consideration of both of the aforementioned contamination sources - that from the upper respiratory tract introduced during sampling and that introduced during laboratory processing steps. We have previously shown that protected bronchoscopy offers some protection from upper airway contamination [4]. In the current study, we address the issue of contamination from the laboratory.

The impact of laboratory contamination is typically evaluated through the inclusion of negative control samples (NCS) that are processed through all steps of DNA extraction and library preparation for sequencing alongside the study samples. The approach is not perfect as one may expect to find taxa in the NCS that also belong to the bacterial communities of the sampled site. Researchers are thus faced with a difficult decision with regards to what to do with the information acquired from the NCS. Some groups have removed all taxa identified in NCS from their study samples [4, 6, 7]. Others single out taxa they believe likely represent contaminants [8]. Currently bioinformatic tools are being developed that aim to wriggle out the authentic microbiota signal using statistical models [9–11], but these have yet to be tested on lower respiratory tract sequencing data (e.g. Decontam [9]).

In the current paper we illustrate an effective workflow for evaluating the quality of lower respiratory tract samples for accurate assessment of bacterial composition. Objectives of the study were i) to determine the influence of contamination on lower respiratory tract samples as a function of bacterial load, ii) to determine the main source of contamination in our laboratory setting and iii) to explore common in silico approaches to dealing with contamination.

## Results

In order to establish the bacterial load in protected airway samples collected using different sampling techniques, we included oral washes (OW), two fractions of protected bronchoalveolar lavage (PBAL1 and PBAL2) and protected specimen brushes (PSB) from 23 participants of the MicroCOPD study [12]. The subject characteristics are provided in Table 1.

**Table 1:** Subject characteristics

	Controls	COPD	Asthma
Subjects	9	10	4
Age	63.0 ± 6.7	68.2 ± 5.2	63.6 ± 3.1
Men	6 (66.7%)	8 (80.0%)	2 (50.0%)
Current-smokers	2 (22.2%)	1 (10.0%)	0
Former-smokers	5 (55.6%)	9 (90.0%)	3 (75.0%)
Non-smokers	2 (22.2%)	0	1 (25.0%)
Smoker pack years	11.8 ± 6.1	25.2 ± 8.1	12.1 ± 6.2
FEV_1_ (% predicted)	97.0 ± 13.7	72.6 ± 23.2	101.6 ± 9.3
Inhaled corticosteroids	0	2 (20.0%)	3 (75.0%)
LABA	0	3 (30.0%)	1 (25.0%)
LAMA	0	4 (40.0%)	0

*COPD* chronic obstructive pulmonary disease, *FEV*_*1*_ forced expiratory volume in 1 s, *LABA* long-acting beta-agonist, *LAMA* long-acting muscarinic antagonist. 1 smoker pack year = 20 cigarettes (one pack) smoked daily for 1 year. Age, smoker pack years and FEV_1_ (% predicted) are presented as the mean ± standard deviation

### Bacterial load varies with sample type

**Fig. 1:**
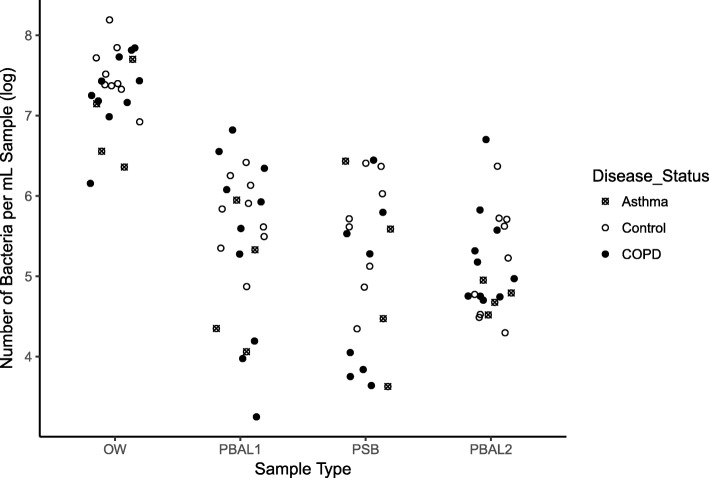
Measured bacterial load in procedural samples (OW, PBAL1, PSB and PBAL2). The mean bacterial load in OW samples was approximately 30 fold higher than PBAL1, 50 fold higher than PSB and, 70 fold higher than PBAL2. OW: oral wash (*n* = 23); PBAL1: first fraction of protected BAL from right middle lobe (*n* = 23); PSB: protected specimen brush from right lower lobe (*n* = 20); PBAL2: second fraction of protected BAL from right middle lobe (*n* = 23)

The bacterial load in the four sample types collected per subject was measured by probe based quantitative PCR (qPCR) targeting the bacterial 16S rRNA gene V1 V2 region. The bacterial load decreased in order OW > PBAL1 > PSB > PBAL2 (*p* < 0.001, non-parametric trend test) (Fig. 1). The mean number of bacteria (× 10^6^/ mL sample) was 34.2 (range 1.4 to 155.8) for OW (*n* = 23); 1.1 (range 1.7 × 10^− 3^ to 6.6) for PBAL1 (n = 23); 0.7 (range 4.3 × 10^− 3^ to 2.8) for PSB (*n* = 20) and 0.5 (range 19.9 × 10^− 3^ to 5.1) for PBAL2 (n = 23).

### Bacterial load and impact of laboratory contamination

**Fig. 2:**
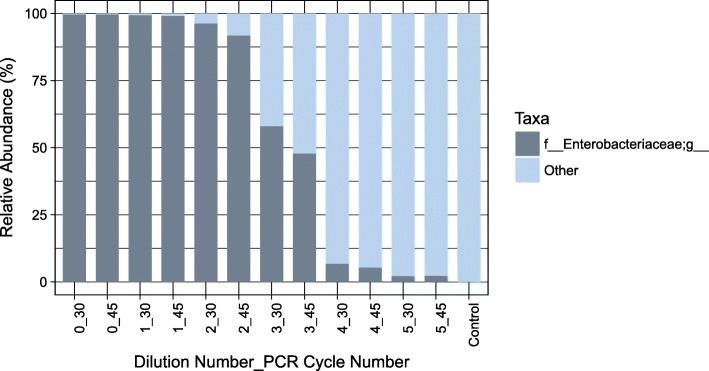
Estimate of contaminant levels in ten-fold dilution series of *Salmonella* (SDS). The major operational taxonomic units (OTUs) observed in the initial *Salmonella* sample (10^7 bacteria/mL) were assigned to *f__Enterobacteriaceae;g__.* Using the NCBI nucleotide BLAST tool we confirmed that these OTUs (OTU821080, OTU813457 and OTU813217) matched to the genus *Salmonella*. With each successive dilution, the relative abundance of *f__Enterobacteriaceae;g__* decreased. By dilution 3 (45 PCR cycles), the percentage had reduced to 47.83%. For comparison, PCR amplification of the 16S rRNA gene was performed at both 30 and 45 cycles for all SDS samples. The control is a sample of PCR water processed through steps of PCR and sequencing alongside the SDS samples. Taxonomic rank is described using prefixes (*f__*: family, *g__*: genus)

Salter and colleagues [5] have previously illustrated the inverse relationship between the bacterial load in a sample and the influence of contamination on the bacterial community readout. Once we had established that the bacterial load varied with sampling technique (Fig. 1), we questioned whether the differences in bacterial load for each of the patient samples would also reflect differences in susceptibility to laboratory contamination. Using the Salter approach [5], we estimated the degree of contamination as a function of bacterial load (Fig. 2), and translated this to an estimate of contamination in the procedural samples (OW, PBAL, PSB). Using quantitative PCR we determined that the initial *Salmonella* sample had a concentration of 10^7^ bacterial cells/mL. As expected the oral wash samples having a high bacterial load (mean of approximately 10^7^ bacterial cells/mL), will not be greatly impacted by contamination. Samples from the lungs (PBAL, PSB) fell between dilution 2 and 3 (Fig. 2), with contamination representing 10–50% of the bacterial community readout. The impact of varying number of PCR cycles was low (Fig. 2).

### Monitoring procedural contamination

Having learned that contaminating bacterial DNA likely represents a substantial proportion (10–50%) of the sequencing output for the lower airway samples in our study, we attempted to identify the main contamination source. We performed ten simulated bronchoscopy procedures (no patient) over two days to capture the environmental contaminants that may have been introduced during sampling.

All procedural control samples were sequenced together on the same sequencing run (Run A). Additional control samples were sequenced on a second run (Run B) and included samples of molecular grade water that were processed through the DNA extraction protocol without the introduction of PBS. Although sequenced on a separate sequencing run (Run B), the molecular grade water samples would indicate whether the PBS was the main source of contamination. A sample of molecular grade water that was not processed through the DNA extraction protocol (PCR water) was also included on both sequencing runs (Run A and B). This later sample would reflect contamination introduced during PCR and sequencing steps without interference from contamination introduced during sampling and DNA extraction steps.

**Fig. 3:**
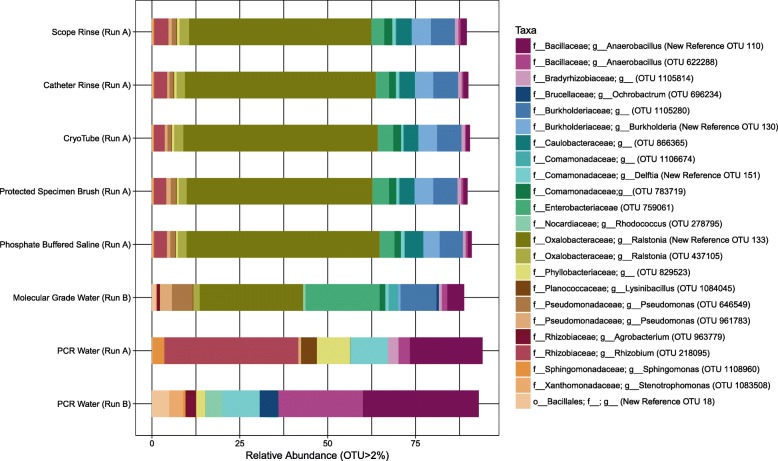
Distribution of operational taxonomic units (OTUs) in procedural controls and PCR water samples. An OTU belonging to the genera *Ralstonia* dominated the procedural control samples with an average relative abundance of 51.81% in scope rinse (*n* = 10), 54.33% in catheter rinse (n = 10), 55.36% in cryotube (*n* = 9), 52.82% in protected specimen brushes (n = 10) and 54.93% in phosphate buffered saline (n = 10). The same *Ralstonia* OTU also dominated the molecular grade water samples (*n* = 3) at an average relative abundance of 29.42%. The PCR water control sample was dominated by *Rhizobium* (38.11%), *Anaerobacillus* New Reference OTU 110 (20.69%) and *Delftia* (10.65%) in run A and *Anaerobacillus* New Reference OTU 110 (32.93%), *Anaerobacillus* OTU 622288 (24.04%) and *Delftia* (10.68%) in run B. Taxonomic rank is described using prefixes (*o__*: order, *f__*: family, *g__*: genus). Data unrarefied

The total number of sequences obtained from the procedural control samples (Run A) after quality filtering and chimera removal was 4.8 × 10^6^. The mean number of sequences and operational taxonomic units (OTUs) obtained from the procedural controls were for phosphate buffered saline (*n* = 10): 64,745 sequences (123 OTUs); catheter rinse (n = 10): 98,379 sequences (131 OTUs); protected specimen brushes (n = 10): 106,853 sequences (132 OTUs); bronchoscope rinse (n = 10): 109,765 sequences (134 OTUs); cryotube (*n* = 9): 115,633 sequences (138 OTUs). The number of sequences obtained from the PCR water control sequenced on the same run (Run A) was lower than for the procedural control samples with only 43,433 sequences and 65 OTUs, suggesting that contamination was predominantly introduced prior to PCR steps of library preparation. The procedural control samples (Run A) showed a similar taxonomic distribution that was quite distinct from that of the PCR water sample (Run A) (Fig. 3). This indicated that contamination was either introduced with the phosphate buffered saline used for collection of all samples or during DNA extraction steps.

To differentiate between PBS and DNA extraction as contamination sources, we compared the molecular grade water samples (Run B) to the corresponding PCR water sample sequenced on the same run. The molecular grade water (*n* = 3) (Run B) contained a mean number of 124,941 sequences and 107 OTUs, whereas the PCR water (Run B) contained 126,103 sequences and only 39 OTUs. Importantly, the taxonomic profile of the molecular grade water (Run B) resembled that of the procedural control samples (Run A), whereas the PCR water did not, indicating that the main source of contamination was the DNA extraction kit (Fig. 3).

### Exploring in silico approaches to dealing with contamination in LRT samples

**Fig. 4:**
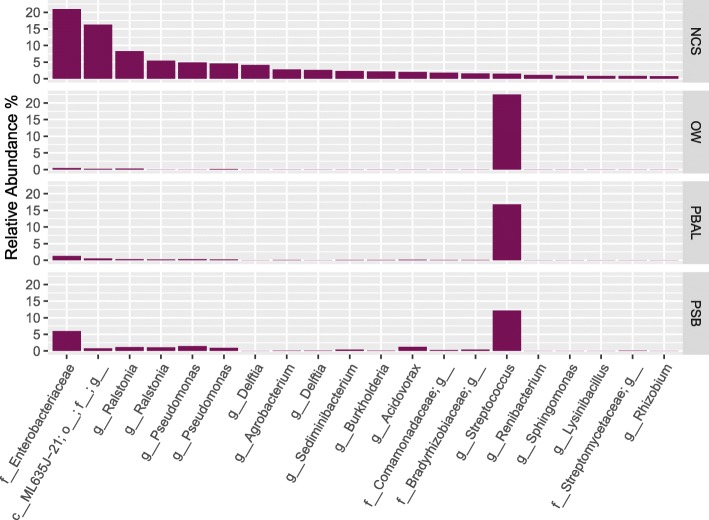
Distribution of the 20 most abundant operational taxonomic units (OTUs) observed in negative control samples (NCS). The NCS were dominated by OTU 759061 assigned to the family *Enterobacteriaceae* (20.93%), OTU 4389128 assigned to a genus within the class *ML635J-21* (16.31%), OTU 437105 and New. Reference OTU 133 both assigned to the genus *Ralstonia* (8.30 and 5.45%, respectively). Taxonomic rank is described using prefixes (*c__*: class, *o__*: order, *f__*: family, *g__*: genus). Data presented as the average relative abundance. Data unrarefied

We began our analyses by looking at how the top 20 OTUs present in NCS were distributed in the procedural samples (OW, PBAL, PSB) in our 23 subjects (Fig. 4). The NCS were dominated by an OTU that mapped to the family *Enterobacteriaceae*. The *Ralstonia* OTU that dominated the procedural controls (Fig. 3) was the fourth most abundant OTU in the NCS with an average relative abundance of just 5.45%. This likely reflects differences in contamination introduced from different lots of the FastDNA Spin Kit [5]. An OTU assigned to the *Streptococcus* genus was found in NCS at a relative abundance of just 1.51%; the same OTU was a major OTU in patient OW, PBAL and PSB samples. This is most likely not a contaminant and may be an important component of the bacterial lung microbiota. For a detailed presentation of the *Streptococcus* OTUs found in PSB and NCS samples, see Additional file 1: Figure S1 and Additional file 2: Figure S2.

**Fig. 5:**
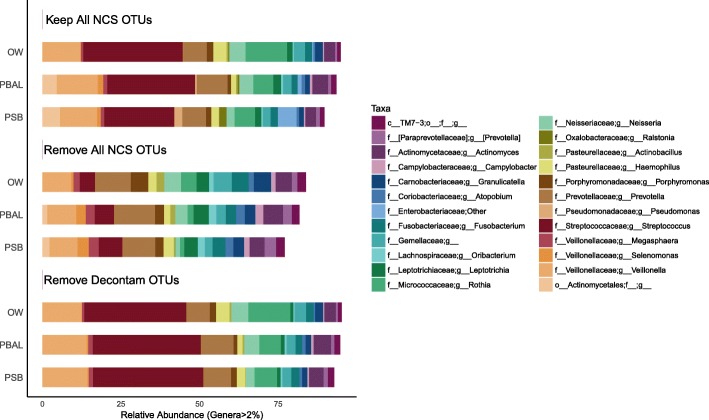
Taxonomic distribution in procedural samples when different approaches to dealing with contamination have been applied. When negative control sample (NCS) operational taxonomic units (OTUs) are kept, the *Streptococcus* genus dominated the procedural samples with an average relative abundance of 31.66% in oral wash (OW) (*n* = 23), 27.95% in protected bronchoalveolar lavage (PBAL) (n = 23) and 22.27% in protected specimen brushes (PSB) (n = 23). With the removal of NCS OTUs, the *Streptococcus* genus no longer dominated the procedural samples and was present at an average relative abundance of 4.80% in OW (n = 23), 6.12% in PBAL (n = 23) and 7.60% in PSB (n = 23). With the removal of OTUs identified as contaminants in Decontam (method = “either”, threshold = 0.5), the *Streptococcus* genus again dominated the samples, with an average relative abundance of 32.52% in OW (n = 23), 34.40% in PBAL (n = 23) and 35.08% in PSB (n = 23). Taxonomic rank is described using prefixes (*c__*: class, *o__*: order, *f__*: family, *g__*: genus). Data unrarefied

Common in silico approaches to dealing with contamination include i) leaving the samples intact (i.e. do nothing), ii) removing all OTUs seen in NCS, and iii) correction based on statistical models (i.e. the Decontam R package). We next examined how the application of each approach would impact the taxonomic profiles of the procedural samples in our study (Fig. 5).

**Fig. 6:**
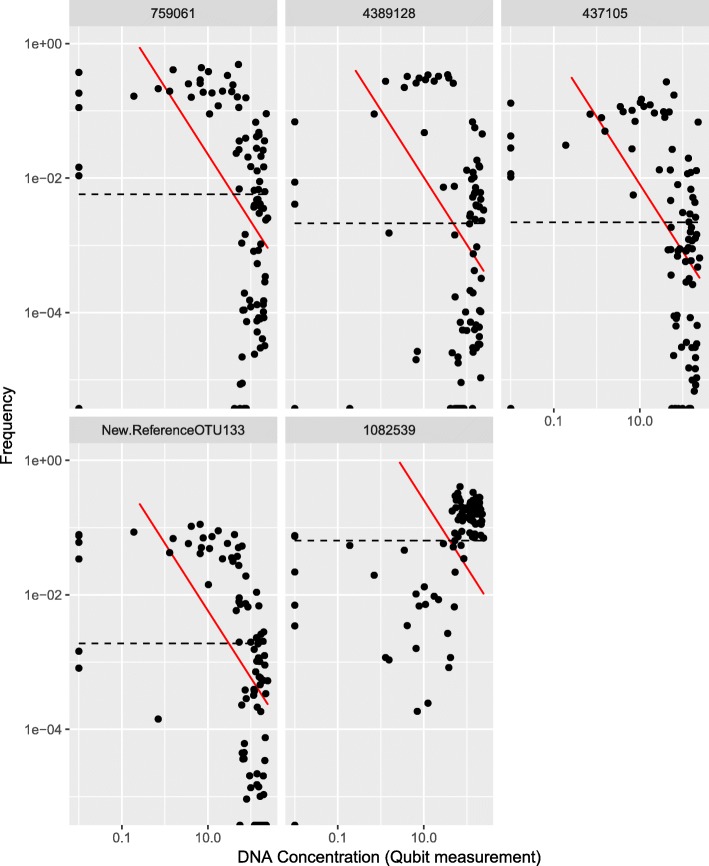
Decontam frequency distribution plots distinguish contaminants from non-contaminants. A frequency distribution plot generated from samples with varying DNA concentration indicates whether a particular sequence fits the Decontam contaminant (red line) or non-contaminant (black stippled line) model. The first four plots represent the top four operational taxonomic units (OTUs) observed in negative control samples (NCS): OTU 759061 is assigned to the family *Enterobacteriaceae*; OTU 4389128 is assigned to a genus within the class *ML635J-21*; OTU 437105 and OTU New. Reference OTU 133 are both assigned to the genus *Ralstonia*. The final plot represents the *Streptococcus* OTU 1082539 that most likely is not a contaminant, although present among the top 20 OTUs found in NCS. Its frequency distribution pattern more closely fits the Decontam non-contaminant model in contrast to the others

When leaving the procedural samples intact, the *Streptococcus* genus dominated all sample types. With the removal of OTUs seen in NCS, the relative abundance of the *Streptococcus* genus was significantly reduced in all sample types (Fig. 5), as was predicted from Fig. 4. With removal of OTUs identified as contaminants using Decontam [9], the *Streptococcus* genus again dominated the procedural samples. This approach thus appeared to provide a good balance between removing all OTUs found in the NCS and leaving intact OTUs present in both NCS and procedural samples. Comparison of the frequency-based distribution plots for the top 4 OTUs observed in NCS and the *Streptococcus* OTU (Fig. 6), visually illustrate how Decontam (here frequency-based method) is able to differentiate between a contaminant OTU and a non-contaminant OTU.

### Decontam performance test on the *Salmonella* dilution series (SDS)

In the Decontam introduction paper [9], the authors illustrate how Decontam is able to diminish the contaminant signal from the serially diluted *Salmonella* datasets published in the Salter paper [5]. As our study also included a *Salmonella* dilution series (SDS), we were able to test the Decontam package tools on sequencing data generated in the context of our laboratory setting after processing through our chosen bioinformatic pipeline.

**Fig. 7:**
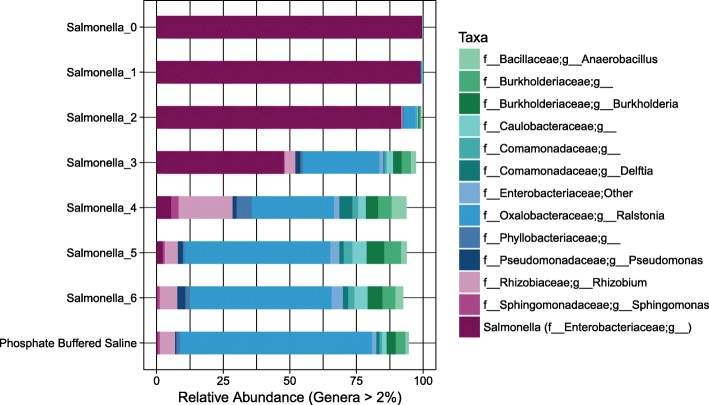
Taxonomic distribution in *Salmonella* dilution series (SDS). The taxonomic profile of the SDS samples (amplified using 45 PCR cycles) before removal of OTUs identified as contaminants in Decontam. Taxonomic rank is described using prefixes (*f__*: family, *g__*: genus). Data unrarefied

The SDS in our study included seven samples of a successively ten-fold diluted *Salmonella* monoculture and a PBS negative control sample that went through DNA extraction and sequencing steps alongside the SDS (Fig. 7). As library preparation for sequencing of the SDS was performed at both 30 and 45 PCR cycles and the impact of varying number of PCR cycles was low (Fig. 2), the sequencing output for both sample sets were used as input in the Decontam analyses. We also included a PCR water control sample that was sequenced on the same sequencing run.

**Fig. 8:**
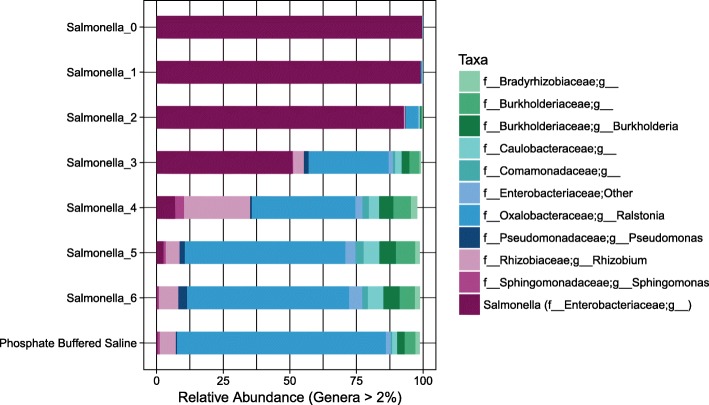
Taxonomic distribution in *Salmonella* dilution series (SDS) after removal of Decontam contaminants (prevalence-based). 109 out of 235 operational taxonomic units (OTUs) in the SDS dataset were identified as contaminants and removed (user defined threshold = 0.5). At the default threshold, only 34 out of 235 OTUs in the dataset were identified as contaminants (figure not drawn). Taxonomic rank is described using prefixes (*f__*: family, *g__*: genus). Data unrarefied

**Fig. 9:**
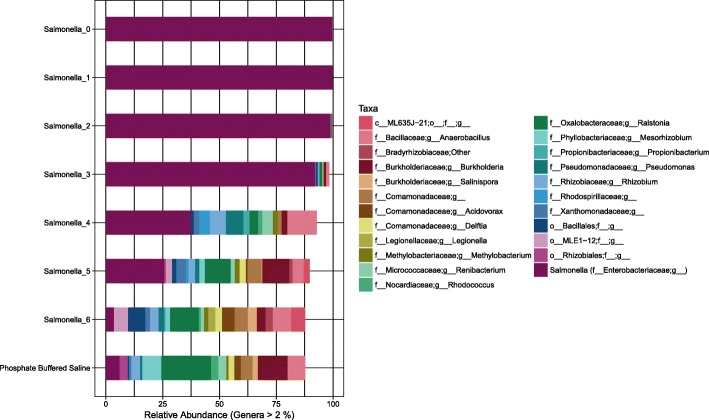
Taxonomic distribution in *Salmonella* dilution series (SDS) after removal of Decontam contaminants (frequency-based). 58 out of 235 operational taxonomic units (OTUs) in the dataset were identified as contaminants and removed (user defined threshold = 0.5). At the default threshold, only 9 out of 235 OTUs in the dataset were identified as contaminants (figure not drawn). Taxonomic rank is described using prefixes (*c__*: class, *o__*: order, *f__*: family, *g__*: genus). Data unrarefied

**Fig. 10:**
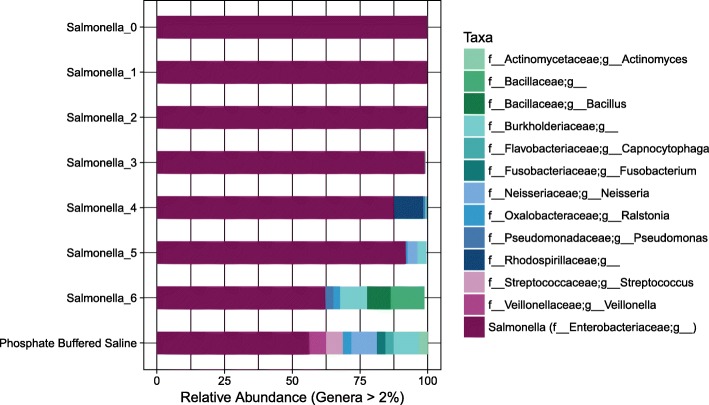
Taxonomic distribution in *Salmonella* dilution series (SDS) after removal of Decontam contaminants (approach either). 136 out of 235 OTUs in the dataset were identified as contaminants and removed (user defined threshold = 0.5). Taxonomic rank is described using prefixes (*f__*: family, *g__*: genus). Data unrarefied

Using the *isContaminant* function in the Decontam R package, we compared three methods for identification of contaminant OTUs including i) the prevalence-based method, ii) the frequency-based method and iii) the either method. In the prevalence-based method, an OTU is marked as a contaminant based on a comparison of how often the OTU is observed in negative control samples compared to the samples under study. For testing the approach on the SDS, the final two samples in the SDS were assigned as negative control samples together with PBS and PCR water samples (as conducted by Decontam developers when testing the approach on the Salter dataset [13]). Figure 8 shows the taxonomic profile of the SDS samples after removal of contaminant OTUs identified using the prevalence-based approach. The impression was that many small OTUs were removed. In the frequency-based approach, the labelling of an OTU as a contaminant is based on the correlation between the DNA concentration measurements made for samples during steps of library preparation (in our lab using the Qubit instrument) and the relative abundance of the OTU across samples. Figure 9 shows the taxonomic profile of the SDS samples after removal of contaminant OTUs identified using the frequency-based approach. The impression was that the frequency-based approach removed fewer but more abundant OTUs compared to the prevalence-based approach. In the final approach tested in Decontam (“either”), all OTUs marked as contaminants by either the prevalence or frequency-based methods are removed (Fig. 10).

Of the three approaches tested in Decontam, the “either” method was able to most effectively remove the contaminant signal from the bacterial community profiles of the samples; even in the most diluted sample over 50% of the sequences mapped to the *Salmonella* genus. Of concern is however that the PBS sample also consisted of over 50% *Salmonella*. Also present in the PBS sample was oral/lung specific genera including *Veillonella*, *Streptococcus* and *Neisseria* that are obvious contaminants from the procedural samples sequenced on the same run. The number of reads in the PBS sample after processing in Decontam was only 32. Therefore we learn that although effective, removal of contaminant OTUs identified in Decontam may also lead to the magnification of another type of noise in the sequencing data – particularly that from cross sample contamination during library preparation or index misassignment during MiSeq sequencing.

## Discussion

In the current paper we illustrate an effective workflow for evaluating the quality of lower airway samples for amplicon-based analysis of bacterial composition. Our results show that the low bacterial load in samples from the lungs make them vulnerable to bacterial DNA contamination, which in our study mainly originated from DNA extraction kits. Even with contaminants representing an estimated 10–50% of the sequencing output for these samples, we demonstrate that most of the contaminating signal can be removed post sequencing using recently developed bioinformatic approaches.

Through the processing and sequencing of a serially diluted culture of *Salmonella* [5], we were able to define the threshold bacterial load for which contamination would begin to dominate the bacterial profile in our samples. At an input of between 10^3 and 10^4 *Salmonella*/mL, we observed that contaminants constituted more than 50% of the bacterial profile of the sample. The use of alternative protocols for sample processing and sequencing can slide this defined threshold of bacterial load up or down and should therefore be determined independently in separate studies. Biesbroek et al. [14] for example show in their study how the choice of DNA extraction kit will affect the DNA yield and in turn the placement of samples above or below a defined threshold of bacterial load for which contamination becomes a problem. Despite differences in laboratory protocols, our results are in agreement with Salter and colleagues [5] who in their study also recommend an input of more than 10^3–10^4 bacterial cells. The concordance of our results may partially be explained by the use of a DNA extraction kit from the same manufacturer (FastDNA Spin Kit, MP Biomedicals).

Using the *Salmonella* dilution series as a reference we were able to determine the degree of laboratory contamination in the various sample types (OW, PBAL1, PBAL2, PSB) collected from participants in the MicroCOPD study. The average bacterial load in the samples acquired from the lungs was highest for PBAL1 samples (10^6 bacteria/mL) and approximately an order of magnitude lower for PSB and PBAL2 samples. This could mean that the first lavage fraction harvests a larger portion of the resident microbiota, but also a dilution effect, as lavage yield tends to increase in the second fraction. We used a sterile inner catheter for lavage sampling, to minimize contamination from BAL, something no other study has done to our knowledge. It is however possible that the first fraction of lavage (PBAL1) is more susceptible to contamination from the upper airways during sampling compared to PBAL2 and PSB samples [4]. Thus, the question remains as to whether PBAL1 with its higher bacterial load is a more representative sample compared to PBAL2 and PSB samples or if we are simply swapping contamination sources (contaminating bacterial DNA introduced from the upper airways during sampling versus contaminating bacterial DNA introduced during laboratory processing steps). The optimal sample type may thus be a question of which contamination source is easiest to identify and remove post sequencing.

Through the sequencing of procedural control samples and PCR negative control samples that were not processed through the DNA extraction protocol, we were able to trace the main source of contamination back to the DNA extraction kit. Our findings are in agreement with several other studies [5, 15, 16]. The difference in the microbiota readout for the procedural control samples and the negative control samples are likely explained by differences in lot number for the DNA extraction kits. Salter and colleagues report differences in contaminant profiles for three replicates of SDS extracted using different lots of the FastDNA Spin Kit for soil; similar to our results they also found that one SDS replicate was dominated by unclassified *Enterobacteriaceae*.

Publications such as that by Salter and colleagues have led to an increased awareness of the effects of contamination on microbiome studies of low biomass samples [5, 16]. Most studies now process negative control samples that allow for monitoring of the contaminant signal introduced from the laboratory. However, the inclusion of NCS only partly addresses the issue. In our study for example, we recognized that a major *Streptococcus* OTU found in procedural samples (OW, PBAL, PSB) was also among the top 20 most abundant OTUs found in NCS. A comparison of the relative abundance of the *Streptococcus* OTU in procedural samples and NCS indicated that the OTU was likely not a contaminant. However, the question of where to draw the line with regards to a set abundance threshold for which an OTU should be identified as a contaminant or not is not always as straightforward. The Decontam package in R has been developed to identify contaminants using statistical models [9]. The Decontam developers demonstrate the accuracy of their approach on the *Salmonella* dilution series datasets generated in the Salter publication. We show in the context of our laboratory setting that Decontam is efficient at removing the contaminant signal from the SDS also in our study. Using Decontam we were also able to confirm the identity of the *Streptococcus* OTU found in both procedural samples and the NCS as a non-contaminant.

We acknowledge that our study does not address all issues related to bacterial load in microbiome sequencing data. The serial diluted *Salmonella* monoculture does not provide insight into the effects of bacterial load on the relative abundance of bacteria in a more complex microbiota sample. Biesbroek et al. [14] show in their study examining the microbiota of a serially diluted saliva sample, an increase in the relative abundance of *Proteobacteria* and *Firmicutes* and a decrease in *Bacteroidetes* across the dilution series. *Proteobacteria* likely reflect contaminants as has been suggested in several papers [14, 17], again illustrating the inverse relationship between bacterial load and the influence of contamination as observed in our study. The observed increase in relative abundance of *Firmicutes* and concurrent decrease in *Bacteroidetes* is however of concern, as these phyla hold members often detected in studies of the lung microbiome (e.g. *Veillonella* and *Prevotella*). The field would benefit from studies addressing the potential effects of bacterial load on the measured relative abundance of taxa in a more complex sample, particularly those that are suspect core lung microbiota members. Secondly, we did not quantify the amount of human DNA in the procedural samples. The presence of human DNA may affect the efficiency of the qPCR reaction [16], and thereby also the accuracy of the direct comparison to the SDS. Studies evaluating the impact of contamination might consider quantification of human DNA for an even more accurate estimate of contamination.

## Conclusions

Measured amounts of bacteria will vary in lower airway samples collected with different bronchoscopic sampling techniques (e.g. PBAL1, PBAL2, PSB in the current study). These differences combined with the inverse relationship between bacterial load and bacterial DNA contamination will render some sampling modalities dominated by contaminating taxa.

Differences in protocols for sampling, laboratory processing and bioinformatics analysis across studies will require investigators to evaluate the impact of contamination in the context of their own laboratory setting. We encourage investigators to report an estimate of the degree of contamination in their datasets defined against a sample of known bacterial load as exemplified in the current study. We further suggest the use of contaminant identification tools (e.g. Decontam) based on statistical models for the objective removal of laboratory contaminants in lung microbiome sequencing data. Such measures will enable more accurate inter-study comparisons and may also resolve discrepancies between studies that have likely impeded understanding the potential relationship between microbiota and its role in chronic lung diseases.

## Methods

### Study samples

Study subjects (*n* = 23) were chosen from the Bergen COPD Microbiome Study (short name “MicroCOPD”) [12], to give an equal representation of healthy (*n* = 9) and diseased (asthma (*n* = 4), COPD (*n* = 10)) states. Details on data collection and the bronchoscopy procedures have been previously published [4, 12]. Briefly, adult subjects recruited from Western Norway with and without obstructive lung disease, underwent voluntary bronchoscopies between 2013 and 2015. All subjects were examined in the stable state, not having received antibiotics at least 2 weeks prior to the procedure. All bronchoscopies were performed by experienced chest physicians at the outpatient clinic at the Department of Thoracic Medicine, Haukeland University Hospital. The regional ethical committee (REK-Nord, case # 2011/1307) approved the study, and all patients gave written informed consent.

Sample types acquired per patient included the first and second fraction of 2 × 50 mL bronchoalveolar lavage (PBAL1 and PBAL2) sampled through a sterile inner catheter (Plastimed Combicath, Le Plessis Bouchard, France) of the bronchoscope while the scope itself was wedged in the right middle lobe, and three protected specimen brushes subsequently sampled from the right lower lobe (rPSB), an oral wash (OW), and a negative control sample (NCS). Additional procedural control samples were collected after ten simulated bronchoscopy procedures (no patient) carried out over two days; samples included a bronchoscope rinse (BR), a catheter rinse (CR), a protected specimen brush (PSB), a sample of phosphate buffered saline (PBS) transferred to a cryotube (CT) and a sample of PBS used for collection of all samples. The PBS used for sample collection was sterilized by sterile filtration (0.22 μm) and autoclaving at 121 °C for 15 min. To study the relationship between bacterial load and the influence of contaminating bacterial DNA in our laboratory setting [5], we included a ten-fold dilution series of *Salmonella enterica serovar Typhimurium* (ATCC 14028) (ATCC, Manassas, VA, USA) (SDS).

### Bacterial DNA extraction using enzymatic and mechanical lysis steps

Samples were treated with lytic enzymes mutanolysin, lysozyme and lysostaphin (all from Sigma-Aldrich, St. Louis, MO, USA) and subsequently processed through the FastDNA Spin Kit (MP Biomedicals, LLC, Solon, OH, USA) following the manufacturer’s instructions. Procedural samples were processed using different lots of the DNA extraction kit (#79113, #84562, #57212, #62903). The procedural controls and the SDS were processed using a kit of same lot number (#93678). The sample volume used as input varied with sample type (for procedural samples: 450 μl for PSB and NCS and 1800 μl for OW, PBAL1, PBAL2; for procedural control samples: 450 μl for PBS and CT, 550 μl for PSB and 1800 μl for BR and CR; for samples in the SDS: 500 μl). DNA was eluted in a total volume of 100 μl.

### Quantification of bacterial load by quantitative PCR (qPCR)

The bacterial load in the samples was determined by probe-based qPCR targeting the bacterial 16S rRNA gene (region V1 V2) using forward primer 5′-AGAGTTTGATCCTGGCTCAG-3′, reverse primer 5′-CTGCTGCCTYCCGTA-3′ and probe 5′-6-FAM-TAACACATGCAAGTCGA-BHQ-1-3′ (locked nucleic acid bases are underlined; 6-FAM: 6-carboxyfluorescein; BHQ-1: Black Hole Quencher-1) [7, 18–20]. PCR reactions were carried out using the following cycling conditions: an initial cycle at 95 °C for 5 min followed by 45 cycles of 95 °C for 5 s, 60 °C for 20 s and 72 °C for 10 s and a final extension cycle of 72 °C for 2 min. A standard curve was constructed from genomic DNA from *E. coli* strain JM109 (Zymo Research, Irvine, CA, USA).

### MiSeq sequencing of the bacterial 16S rRNA gene

The bacterial composition in the samples was determined by paired-end sequencing of the 16S rRNA gene (region V3 V4) following instructions provided in the Illumina 16S Metagenomic Sequencing Library Preparation guide (Part no. 15044223 Rev. B). PCR cycling conditions were modified from the commercial protocol and consisted of an initial cycle at 95 °C for 3 min followed by 45 cycles of 95 °C for 30 s, 55 °C for 30 s, 72 °C for 30 s and a final extension cycle at 72 °C for 5 min.

### Bioinformatic sequence processing steps

Bioinformatic sequence processing steps were performed using tools provided within the Quantitative Insights into Microbial Ecology (QIIME) bioinformatic package, version 1.9.1. In short, raw sequences were retrieved from the MiSeq sequencer in the form of demultiplexed forward and reverse fastq files (paired end reads). Primer sequences were trimmed off and forward and reverse reads joined. Chimera sequences identified using the VSEARCH program [21] were subsequently removed. Remaining sequences were grouped into open-reference operational taxonomic units (OTUs) using UCLUST [22] and the GreenGenes reference database (v.13.8) [23]. Small OTUs, defined as those containing less than 0.005% of the total sequence count in the dataset were then filtered out [24]. Taxonomy was assigned to OTUs using the naïve bayesian RDP Classifier [25] together with the GreenGenes reference database (v.13.8) [23]. The resulting OTU table displaying the sequence count in each OTU for each sample was the starting point for all subsequent analyses. The QIIME commands used for generating the working OTU table are provided in the Additional file 3: Supplementary Methods.

### In silico contaminant identification and removal

Two approaches to contaminant identification and subsequent removal were tested. In the first approach contaminant OTUs were identified through their presence in NCS. NCS OTUs were filtered out from the procedural samples (OW, PSB, PBAL) collected under the same procedure using QIIME commands (illustrated in the supplementary methods). In the second approach, contaminant OTUs were identified based on statistical models using the Decontam package [9] in R. Contaminant OTUs identified using the Decontam *isContaminant* function (method = either, user defined threshold = 0.5) were filtered out of the main OTU working table using QIIME commands.

For greater details on study design, sample collection, preparation of *Salmonella* samples, DNA extraction, qPCR, 16S rRNA gene sequencing and bioinformatics, please see the Additional file 3: Supplementary Methods.

## Additional files

Additional file 1: **Figure S1.** Distribution of *Streptococcus* OTUs in Protected Specimen Brush (PSB) samples (*n*=23). (PDF 8 kb)

Additional file 2: **Figure S2.** Distribution of *Streptococcus* OTUs in Negative Control Samples (NCS) (n=23). (PDF 8 kb)

Additional file 3: Supplementary Methods. This file provides a detailed description of protocols for sample collection, preparation of *Salmonella* samples, DNA extraction, qPCR, 16S rRNA gene sequencing and bioinformatics. (DOCX 240 kb)

## Data Availability

The fastq files and metadata needed to rerun the analyses performed in the current study will be available in the DRYAD repository upon publication (doi:10.5061/dryad.1v92t8b). Bioinformatics analyses steps are described in the Additional file 3: Supplementary Methods. A detailed description of the protocols and laboratory materials used in the MicroCOPD Study are available at dx.doi.org/10.17504/protocols.io.2sygefw.

## Abbreviations

COPD: Chronic obstructive pulmonary disease
NCS: Negative control sample
OTU: Operational taxonomic unit
OW: Oral wash
PBAL: Protected bronchoalveolar lavage
PSB: Protected specimen brush
QIIME: Quantitative Insights into Microbial Ecology
qPCR: Quantitative PCR
