# Supplementary figures and images for: Laboratory contamination in airway microbiome studies
Source: BMC Microbiol. 2019 Aug 14;19:187. doi: 10.1186/s12866-019-1560-1 (PMC6694601; doi:10.1186/s12866-019-1560-1)

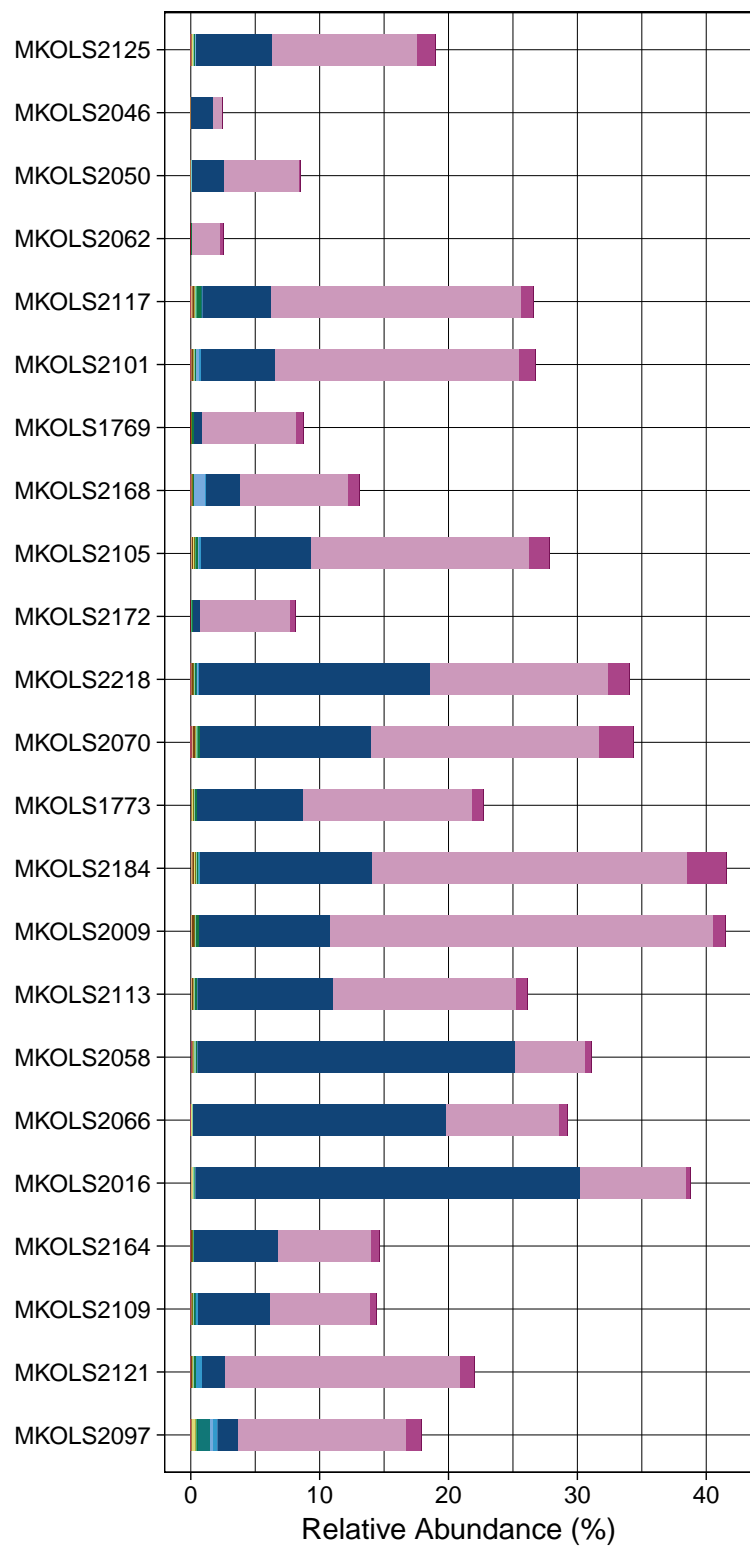

Supplement: Supplementary file 1 — Figure S1. Distribution of Streptococcus OTUs in Protected Specimen Brush (PSB) samples (n=23). (PDF 8 kb) [file 12866_2019_1560_MOESM1_ESM.pdf]

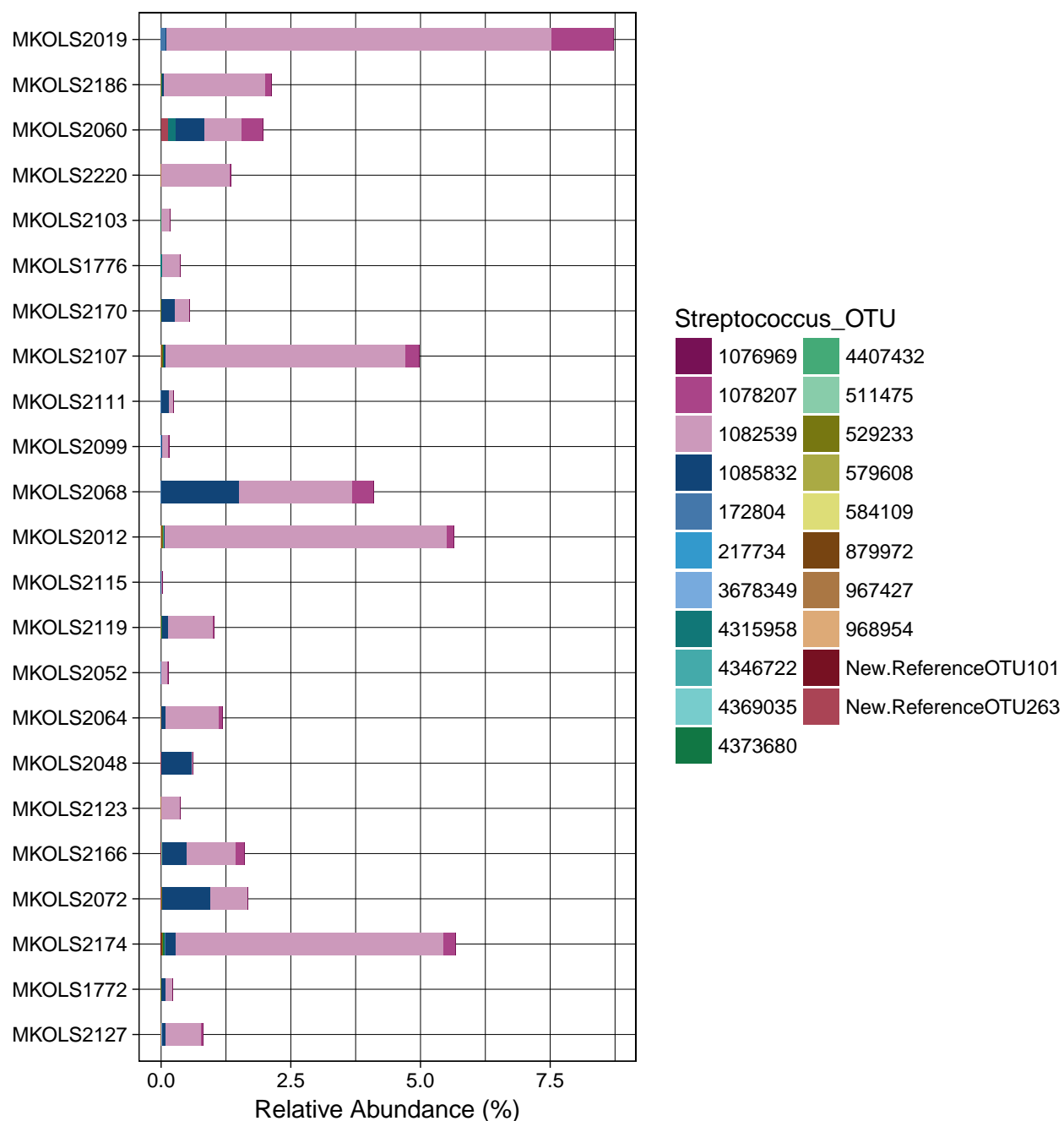

Supplement: Supplementary file 2 — Figure S2. Distribution of Streptococcus OTUs in Negative Control Samples (NCS) (n=23). (PDF 8 kb) [file 12866_2019_1560_MOESM2_ESM.pdf]
