# Supplementary material for: Laboratory contamination in airway microbiome studies
Source: BMC Microbiol. 2019 Aug 14;19:187. doi: 10.1186/s12866-019-1560-1 (PMC6694601; doi:10.1186/s12866-019-1560-1)
Supplement: Supplementary file 3 — Supplementary Methods. This file provides a detailed description of protocols for sample collection, preparation of Salmonella samples, DNA extraction, qPCR, 16S rRNA gene sequencing and bioinformatics. (DOCX 240 kb) [file 12866_2019_1560_MOESM3_ESM.docx]

**Supplementary Methods**

The study was approved by the regional medical ethics committee (project number 2011/1307) and was conducted in accordance with the Declaration of Helsinki. All study subjects signed informed consent forms.

**Design**

We performed quantitative PCR and high-throughput sequencing of the 16S rRNA gene V3V4 region for analysis of bacterial communities in control samples and bronchoscopic, procedural samples collected from 23 subjects from the Bergen COPD Microbiome study [1]. We evaluated the influence of laboratory contamination on the bacterial community profiles as a function of bacterial load through sequencing of a ten fold diluted mono-culture of Salmonella (approach used by [2]). Finally, we explored three different in silico approaches to dealing with contaminating bacterial DNA in lung microbiome sequencing data.

**Sample Collection: Procedural Samples**

The bronchoscopy procedure has been described previously in detail [3]. Briefly, a flexible video-bronchoscopy was performed in supine position using oral access, on mildly sedated subjects (alfentanil). Local anaesthesia was achieved with pre-procedural lidocaine spray and per-operative lidocaine through a catheter. No suction was allowed before reaching the carina. Sample types acquired per patient included the return of the first and second fraction of protected bronchoalveolar lavage (PBAL1 and PBAL2) from the right middle lobe, 3 protected specimen brushes from the right lower lobe (PSB), the return of 10 ml phosphate-buffered saline (PBS) oral wash (OW) and a negative control sample (NCS).

**Sample Collection: Procedural Control Samples**

The team that had been responsible for sampling of patients in the MicroCOPD study returned to the clinic and set up a scenario of ten simulated (no patient) bronchoscopy procedures over two days with the aim of capturing the environmental contaminants that may have been introduced during sampling of patients. On each day, a fresh 500 mL bottle of PBS was opened and used during sample collection for all five procedures carried out. For each procedure, five negative control samples were collected and included a bronchoscope rinse (BR), a catheter rinse (CR), a protected sampling brush (PSB), a sample of PBS transferred to a cryotube (CT) and a sample of the phosphate buffered saline (PBS) used for collection of all samples. After each procedure the flexible bronchoscope was cleaned as during patient sampling in an endoscope washer-disinfector (Wassenburg WD440) and left to dry in a drying cabinet. Four bronchocopes were used for the procedures, with serial numbers 2501807, 2501834, 2501803, 2501838.

**Preparation of Salmonella Samples**

Salmonella typhimurium (ATCC 14028) was plated out on blood agar plates and incubated overnight at 37 °C. Colonies were transferred to a tube containing 3 mL sterile physiological water using sterile cotton swabs until a Macfarland Density of approximately 4 was reached. This starter suspension was then used to create a 10 fold dilution series across a total of seven samples. DNA was extracted using the same protocol as for the procedural and procedural control samples.

**Bacterial DNA Extraction Using Enzymatic and Mechanical Lysis Steps**

The sample volume used as input for DNA extraction varied with sample type – 1800 µl for OW and PBAL and 450 µl for PSB and NCS. For procedural control samples input sample volume was 1800 µl for BR and CR, 550 µl for PSB and 450 µl for CT and PBS. For the SDS an input volume of 500 µl was used. Prior to bacterial cell lysis steps, the sample was treated with Sputasol (Oxoid) to disrupt mucin disulfide bonds and ensure a homogenous distribution of the bacteria in the sample. The volume of Sputasol added was equal to the input sample volume for DNA extraction (1:1 ratio). After addition of Sputasol, the sample was incubated at 37 °C for 15 minutes on a thermomixer (1000 rpm). The bacterial cells were then collected by centrifugation at 15700 g for 8 minutes and resuspended in 250 µl PBS. Next the sample was treated with an enzyme cocktail solution consisting of 25 µl lysozyme (10 mg/mL, Sigma-Aldrich), 3 µl mutanolysin (25 KU/mL, Sigma-Aldrich), 1.5 µl lysostaphin (4000 U/mL, Sigma-Aldrich) and 20.5 µl TE5 buffer (10 mM Tris-HCl, 5 mM EDTA, pH 8) and incubated at 37 °C for 1 hour on a thermomixer (350 rpm). Any bacterial cells not sufficiently lysed by the enzymes were collected by centrifugation at 15700 g for 15 minutes. The supernatant containing any extracted bacterial DNA was transferred to a new eppendorf tube and stored at 4 °C while further processing of the hard to lyse bacterial cell pellet. The pellet was resuspended in 800 µl CLS-TC lysis buffer (FastDNA Spin Kit, MP Biomedicals, LLC, Solon, OH, USA) and transferred to a Lysing Matrix A tube (FastDNA Spin Kit). The sample was then subjected to mechanical lysis using the FastPrep-24 instrument (MP Biomedicals, LLC, Solon, OH, USA) at a speed setting of 6.0 m/s for 40 seconds. The lysate was pooled with the supernatant from the enzyme lysis step and the sample further processed as described by the manufactuerers for the FastDNA Spin Kit. DNA was eluted in a total volume of 100 µl.

**Quantification of Bacterial Load by Quantitative PCR (qPCR)**

The bacterial load in the samples was determined by probe-based quantitative PCR (qPCR) on a LightCycler 480 instrument (Roche). The primer set used targeted the bacterial 16S rRNA V1V2 gene region using sequences 5′-AGAGTTTGATCCTGGCTCAG-3′ (forward) and 5′-CTGCTGCCTYCCGTA-3′ (reverse). The hydrolysis probe with sequence 5´-6-FAM-TAACACATGCAAGTCGA-BHQ-1-3´ (locked nucleic acid nucleotides are underlined) was labelled with 6-carboxyfluorescein (6-FAM) at the 5 prime end and Black Hole Quencher-1 (BHQ-1) at the 3 prime end. A standard curve was constructed from a 10 fold dilution series of genomic DNA from E. coli strain JM109 (Zymo Research E2006-2). Each reaction consisted of 10 µl Takyon No Rox Probe MasterMix (2x) (Eurogentec), 0.2 µl of each forward and reverse primer (10 µM), 0.15 µl of the hydrolysis probe (10 µM), 2 µl PCR-grade water and 2 µl sample. The PCR reaction was carried out using the following cycling conditions: an initial cycle at at 95 °C for 5 minutes followed by 45 cycles of 95°C for 5 seconds, 60°C for 20 seconds and 72°C for 10 seconds and a final extension cycle of 72°C for 2 minutes.

**16S rRNA Gene Library Preparation and Sequencing**

Library preparation and sequencing of the bacterial 16S rRNA gene V3V4 region was performed according to the Illumina 16S Metagenomic Sequencing Library Preparation guide (Part no. 15044223 Rev. B). In brief the protocol consisted of two steps of PCR - an initial amplicon PCR step for amplification of the 16S rDNA V3V4 region and a subsequent index PCR step for the addition of index sequences required for sample multiplexing. The primers used in the initial amplicon PCR step consisted of a gene specific region targeting conserved sequences flanking the 16S rDNA V3V4 region and additional overhang adaptor sequences (underlined) required for downstream library preparation and sequencing steps:

5′-TCGTCGGCAGCGTCAGATGTGTATAAGAGACAGCCTACGGGNGGCWGCAG-3´ (forward) and

5′-GTCTCGTGGGCTCGGAGATGTGTATAAGAGACAGGACTACHVGGGTATCTAATCC-3´ (reverse)

PCR cycling conditions were modified from the commercial protocol and consisted of an initial cycle at 95°C for 3 minutes followed by 45 cycles of 95 °C for 30 seconds, 55 °C for 30 seconds, 72 °C for 30 seconds and a final extension cycle at 72 °C for 5 minutes. The PCR products were visualized by running a 1 % agarose gel. The subsequent Index PCR step was carried out using primers from the Nextera XT Index Kit (Illumina Inc., San Diego. CA, USA) which targeted the adaptor sequences incorporated to the 16S amplicons in the first PCR. The Nextera XT Index kit included 8 forward primers and 12 reverse primers with unique index sequences, enabling multiplexing of up to 96 samples on each sequencing run. The 16S amplicon libraries were quantified using the Qubit dsDNA HS Assay Kit (Life Technologies), normalized to 4nM and pooled together. The samples were prepared for 2 x 300 cycles of paired-end sequencing using reagents from the Miseq reagent kit v3 (Illumina).

**Bioinformatic Sequence Processing Steps**

Sequence processing steps were performed within the Quantitative Insights Into Microbial Ecology (QIIME) bioinformatic package, version 1.9.1. Two fastq files per sample were retrieved from the Illumina Miseq instrument - one for the forward read and one for the reverse read (paired end reads). The primer sequences were removed by trimming off the first 17 and last 21 bases using the multiple_extract_barcodes.py script. The forward and reverse paired end reads were then joined using the join_paired_ends.py. The default fastq-join method was chosen with a minimum requirement of 100 bases of overlap. Quality filtering was carried out using the multiple_split_libraries.py script. We chose to set the minimum base quality score to a Phred value of 20. This translates to a probability of 1 in 100 that a base is called incorrectly. Chimeras were identified by running the script identify_chimeric_seqs.py using the VSEARCH [4] algorithm and removed. The remaining sequences were clustered into Operational Taxonomic Units (OTUs) using pick_open_reference_otus.py. The script uses the UCLUST clustering algorithm [5] together with the GreenGenes reference database (v.13.8)[6] to group sequences together based on a 97% sequence similarity threshold. Taxonomy was assigned to representative OTU sequences using the naïve bayesian RDP classifer together with the GreenGenes reference database v.13.8) [6]. Finally the OTU table was screened for small OTUs using the filter_otus_from_otu_table.py command with the min_count_fraction flag set to 0.00005. This action removes all OTUs with a sequence count less than 0.005% of the total sequence count in the dataset as recommended in the literature [7]. Analysis of taxonomy was carried out in Excel. Figures were generated in R (r-project.org).

**In Silico Approaches to Dealing with Contamination**

We explore three approaches to dealing with contamination: i) keep all samples intact (i.e. do nothing), ii) remove OTUs found in NCS and iii) remove OTUs identified as contaminants using the Decontam R package [8]. For approach one, analyses were performed on the main working table generated as described in the previous section. A description of the second two approaches follows. An overview of the samples included in the analysis is provided in Table S.1.

**Approach Two: Removal of Contaminant OTUs Identified by Their Presence in NCS**

The main working OTU table described previously was split by subject ID using the split_otu_table.py command. The resulting 23 OTU tables consisted of 4 samples each (OW, PSB, PBAL and NCS). OTUs found in the NCS were removed using QIIME commands as described in Figure S.1. Subsequently the 23 OTU tables (now screened for NCS OTUs and NCS) where merged back together using the merge_otu_tables.py command. Analysis of taxonomy was performed in Excel.

**Approach Three: Removal of Contaminant OTUs Identified Using the Decontam R Package**

Contaminant OTUs were identified in procedural samples (OW, PSB, PBAL and NCS) using the isContaminant function available in the Decontam [8] R package. The possession of both negative controls (NCS/PCR water samples) and DNA quantitation data (qubit measurements) enabled us to take use of the approach “either”, within the isContaminant function. For six samples, the DNA quantitation data (measured during Miseq library preparation steps) fell below the detection limit of the Qubit instrument. Rather than removing these samples from the analysis, we set the DNA concentration to the lower limit of the Qubit instrument (0.01 ng/µl). We set the user defined threshold value to 0.5 (default=0.1). This aggressive classification threshold was chosen for best comparison to approach 2 (remove all OTUs observed in NCS). The output from the isContaminant analysis (method=”either”, threshold=0.5) was a list of all OTUs identified as contaminants by either the “prevalence” or “frequency” based contaminant classification algorithms. The contaminant OTUs were removed from the OTU table using the filter_otus_from_otu_table.py QIIME command. Analysis of taxonomy was performed in excel.

We note that the alternative to the isContaminant function (the isNotContaminant function) is recommended by the developers for samples where the proportion of contaminant sequences > than the proportion of noncontaminant sequences (i.e. for low biomass samples). Having estimated the proportion of contaminants in our procedural samples to be less than 50%, we found the isContaminant function most appropriate.

**Decontam Performance Test on the Salmonella Dilution Series**

We tested the *isContaminant* method on the serially diluted *Salmonella* samples (SDS). The SDS included seven samples of a successively ten-fold diluted *Salmonella* monoculture and a PBS negative control sample that went through DNA extraction and sequencing steps alongside the SDS. As library preparation for sequencing of the SDS was performed at both 30 and 45 PCR cycles and the impact of varying number of PCR cycles was low, the sequencing output for both sample sets were included in the Decontam analysis. We also included a PCR water control sample that was sequenced on the same sequencing run for a total of 17 samples. We assigned the final two samples in the SDS as controls together with the PBS and PCR water samples. DNA concentration measurements were available for all samples except for one negative control PBS sample, which fell below the detection limit on the Qubit instrument. We set the concentration of the sample to the lower detection limt for the instrument (0.01 ng/ul). An overview of the samples included in the analysis is provided in Table S.2.


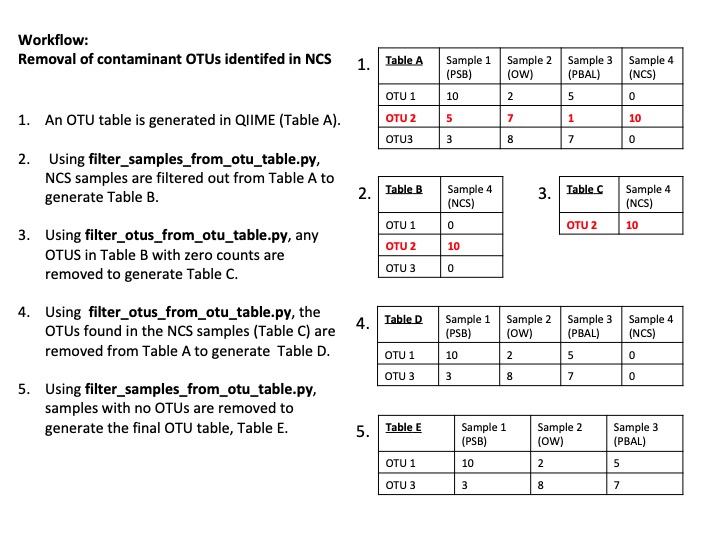


**Figure S.1. QIIME Workflow for removal of NCS OTUs from procedural samples.**

**Table S.1. Procedural samples.**

| Sample ID | Subject ID | Sample Type | Sample or Control | Miseq Run | Qubit (ng/ul) |
| --- | --- | --- | --- | --- | --- |
| MKOLS1769 | MK158 | PSB | Sample | 20 | 62.29 |
| MKOLS1771 | MK158 | OW | Sample | 20 | 77.92 |
| MKOLS1772 | MK158 | NCS | Control | 20 | 41.61 |
| MKOLS1770 | MK158 | PBAL1 | Sample | 20 | 56.76 |
| MKOLS1773 | MK162 | PSB | Sample | 20 | 62.77 |
| MKOLS1775 | MK162 | OW | Sample | 20 | 55.56 |
| MKOLS1776 | MK162 | NCS | Control | 20 | 0.00* |
| MKOLS1774 | MK162 | PBAL1 | Sample | 20 | 69.50 |
| MKOLS2009 | MK185 | PSB | Sample | 22 | 53.50 |
| MKOLS2011 | MK185 | OW | Sample | 22 | 69.30 |
| MKOLS2012 | MK185 | NCS | Control | 22 | 0.19 |
| MKOLS2014 | MK185 | PBAL2 | Sample | 22 | 61.30 |
| MKOLS2016 | MK189 | PSB | Sample | 22 | 52.30 |
| MKOLS2018 | MK189 | OW | Sample | 22 | 65.10 |
| MKOLS2019 | MK189 | NCS | Control | 22 | 0.00* |
| MKOLS2021 | MK189 | PBAL2 | Sample | 22 | 45.60 |
| MKOLS2046 | MK198 | PSB | Sample | 23 | 51.23 |
| MKOLS2047 | MK198 | OW | Sample | 23 | 93.55 |
| MKOLS2048 | MK198 | NCS | Control | 23 | 7.05 |
| MKOLS2049 | MK198 | PBAL2 | Sample | 23 | 75.28 |
| MKOLS2050 | MK199 | PSB | Sample | 23 | 28.50 |
| MKOLS2051 | MK199 | OW | Sample | 23 | 99.33 |
| MKOLS2052 | MK199 | NCS | Control | 23 | 1.55 |
| MKOLS2053 | MK199 | PBAL2 | Sample | 23 | 119.05 |
| MKOLS2058 | MK204 | PSB | Sample | 23 | 73.35 |
| MKOLS2059 | MK204 | OW | Sample | 23 | 79.37 |
| MKOLS2060 | MK204 | NCS | Control | 23 | 0.00* |
| MKOLS2061 | MK204 | PBAL2 | Sample | 23 | 52.67 |
| MKOLS2062 | MK205 | PSB | Sample | 23 | 53.39 |
| MKOLS2063 | MK205 | OW | Sample | 23 | 70.47 |
| MKOLS2064 | MK205 | NCS | Control | 23 | 6.59 |
| MKOLS2065 | MK205 | PBAL2 | Sample | 23 | 85.14 |
| MKOLS2066 | MK206 | PSB | Sample | 23 | 76.24 |
| MKOLS2067 | MK206 | OW | Sample | 23 | 121.45 |
| MKOLS2068 | MK206 | NCS | Control | 23 | 0.00* |
| MKOLS2069 | MK206 | PBAL2 | Sample | 23 | 135.16 |
| MKOLS2070 | MK211 | PSB | Sample | 23 | 146.22 |
| MKOLS2071 | MK211 | OW | Sample | 23 | 140.69 |
| MKOLS2072 | MK211 | NCS | Control | 23 | 10.92 |
| MKOL22073 | MK211 | PBAL2 | Sample | 23 | 115.92 |
| MKOLS2097 | MK225 | PSB | Sample | 23 | 120.01 |
| MKOLS2098 | MK225 | OW | Sample | 23 | 139.73 |
| MKOLS2099 | MK225 | NCS | Control | 23 | 1.29 |
| MKOLS2100 | MK225 | PBAL2 | Sample | 23 | 165.95 |
| MKOLS2101 | MK226 | PSB | Sample | 23 | 198.41 |
| MKOLS2102 | MK226 | OW | Sample | 23 | 194.08 |
| MKOLS2103 | MK226 | NCS | Control | 23 | 6.61 |
| MKOLS2104 | MK226 | PBAL2 | Sample | 23 | 171.24 |
| MKOLS2105 | MK227 | PSB | Sample | 23 | 139.25 |
| MKOLS2106 | MK227 | OW | Sample | 23 | 199.86 |
| MKOLS2107 | MK227 | NCS | Control | 23 | 3.52 |
| MKOLS2108 | MK227 | PBAL2 | Sample | 23 | 195.05 |
| MKOLS2109 | MK229 | PSB | Sample | 23 | 210.68 |
| MKOLS2110 | MK229 | OW | Sample | 23 | 184.94 |
| MKOLS2111 | MK229 | NCS | Control | 23 | 38.12 |
| MKOLS2112 | MK229 | PBAL2 | Sample | 23 | 221.74 |
| MKOLS2113 | MK230 | PSB | Sample | 23 | 99.33 |
| MKOLS2114 | MK230 | OW | Sample | 23 | 137.81 |
| MKOLS2115 | MK230 | NCS | Control | 23 | 12.46 |
| MKOLS2116 | MK230 | PBAL2 | Sample | 23 | 136.84 |
| MKOLS2117 | MK232 | PSB | Sample | 23 | 163.78 |
| MKOLS2118 | MK232 | OW | Sample | 23 | 165.70 |
| MKOLS2119 | MK232 | NCS | Control | 23 | 21.89 |
| MKOLS2120 | MK232 | PBAL2 | Sample | 23 | 169.55 |
| MKOLS2121 | MK233 | PSB | Sample | 23 | 150.07 |
| MKOLS2122 | MK233 | OW | Sample | 23 | 151.52 |
| MKOLS2123 | MK233 | NCS | Control | 23 | 4.11 |
| MKOLS2124 | MK233 | PBAL2 | Sample | 23 | 155.84 |
| MKOLS2125 | MK236 | PSB | Sample | 23 | 159.45 |
| MKOLS2126 | MK236 | OW | Sample | 23 | 163.78 |
| MKOLS2127 | MK236 | NCS | Control | 23 | 7.77 |
| MKOLS2128 | MK236 | PBAL2 | Sample | 23 | 152.96 |
| MKOLS2164 | MK241 | PSB | Sample | 24 | 135.40 |
| MKOLS2165 | MK241 | OW | Sample | 24 | 138.77 |
| MKOLS2166 | MK241 | NCS | Control | 24 | 17.39 |
| MKOLS2167 | MK241 | PBAL2 | Sample | 24 | 149.11 |
| MKOLS2168 | MK242 | PSB | Sample | 24 | 117.60 |
| MKOLS2169 | MK242 | OW | Sample | 24 | 227.75 |
| MKOLS2170 | MK242 | NCS | Control | 24 | 35.83 |
| MKOLS2171 | MK242 | PBAL2 | Sample | 24 | 191.68 |
| MKOLS2172 | MK243 | PSB | Sample | 24 | 245.31 |
| MKOLS2173 | MK243 | OW | Sample | 24 | 209.96 |
| MKOLS2174 | MK243 | NCS | Control | 24 | 48.34 |
| MKOLS2175 | MK243 | PBAL2 | Sample | 24 | 215.73 |
| MKOLS2184 | MK094 | PSB | Sample | 24 | 154.16 |
| MKOLS2185 | MK094 | OW | Sample | 24 | 195.53 |
| MKOLS2186 | MK094 | NCS | Control | 24 | 0.70 |
| MKOLS2187 | MK094 | PBAL2 | Sample | 24 | 207.31 |
| MKOLS2218 | MK109 | PSB | Sample | 24 | 129.15 |
| MKOLS2219 | MK109 | OW | Sample | 24 | 197.69 |
| MKOLS2220 | MK109 | NCS | Control | 24 | 10.27 |
| MKOLS2221 | MK109 | PBAL2 | Sample | 24 | 210.68 |
| MKOLS1845 | NA | PCR water | Control | 20 | 0.00* |
| MKOLS2148 | NA | PCR water | Control | 24 | 0.00* |

**Table S.2. Salmonella dilution series (SDS) samples.**

| Sample ID | Sample type | Dilution | Sample_or_Control | PCR cycles | Qubit (ng/ul) |
| --- | --- | --- | --- | --- | --- |
| MKOLS2722 | Salmonella | 0 | Sample | 45 | 34.9 |
| MKOLS2723 | Salmonella | 1 | Sample | 45 | 29.4 |
| MKOLS2724 | Salmonella | 2 | Sample | 45 | 22.2 |
| MKOLS2725 | Salmonella | 3 | Sample | 45 | 15.85 |
| MKOLS2726 | Salmonella | 4 | Sample | 45 | 8.89 |
| MKOLS2727 | Salmonella | 5 | Control | 45 | 9.63 |
| MKOLS2728 | Salmonella | 6 | Control | 45 | 13.75 |
| MKOLS2721 | PBS | NA | Control | 45 | 0.01 |
| MKOLS2730 | Salmonella | 0 | Sample | 30 | 28.2 |
| MKOLS2731 | Salmonella | 1 | Sample | 30 | 18.2 |
| MKOLS2732 | Salmonella | 2 | Sample | 30 | 5.42 |
| MKOLS2733 | Salmonella | 3 | Sample | 30 | 0.34 |
| MKOLS2734 | Salmonella | 4 | Sample | 30 | 0.17 |
| MKOLS2735 | Salmonella | 5 | Control | 30 | 0.28 |
| MKOLS2736 | Salmonella | 6 | Control | 30 | 0.2 |
| MKOLS2729 | PBS | NA | Control | 30 | 0.01* |
| MKOLS2662 | PCR Water | NA | Control | 45 | 0.72 |

**References**

1. Grønseth R, Haaland I, Wiker HG, Martinsen EMH, Leiten EO, Husebø G, et al. The Bergen COPD microbiome study (MicroCOPD): rationale, design, and initial experiences. Eur Clin Respir J. 2014;1.

2. Salter SJ, Cox MJ, Turek EM, Calus ST, Cookson WO, Moffatt MF, et al. Reagent and laboratory contamination can critically impact sequence-based microbiome analyses. BMC Biology. 2014;12:87.

3. Grønseth R, Drengenes C, Wiker HG, Tangedal S, Xue Y, Husebø GR, et al. Protected sampling is preferable in bronchoscopic studies of the airway microbiome. ERJ Open Res. 2017;3.

4. Rognes T, Flouri T, Nichols B, Quince C, Mahé F. VSEARCH: a versatile open source tool for metagenomics. PeerJ. 2016;4. doi:10.7717/peerj.2584.

5. Edgar RC. Search and clustering orders of magnitude faster than BLAST. Bioinformatics. 2010;26:2460–1.

6. McDonald D, Price MN, Goodrich J, Nawrocki EP, DeSantis TZ, Probst A, et al. An improved Greengenes taxonomy with explicit ranks for ecological and evolutionary analyses of bacteria and archaea. ISME J. 2012;6:610–8.

7. Bokulich NA, Subramanian S, Faith JJ, Gevers D, Gordon JI, Knight R, et al. Quality-filtering vastly improves diversity estimates from Illumina amplicon sequencing. Nat Methods. 2013;10:57–9.

8. Davis NM, Proctor D, Holmes SP, Relman DA, Callahan BJ. Simple statistical identification and removal of contaminant sequences in marker-gene and metagenomics data. bioRxiv. 2017;:221499.
